# Patient and Prescriber characteristics associated with return to daily-dispense methadone: A multilevel cohort study

**DOI:** 10.1371/journal.pmen.0000442

**Published:** 2025-11-07

**Authors:** Ria Garg, Shaleesa Ledlie, Daniel McCormack, Nikki Bozinoff, Jennifer Wyman, Beth Sproule, Pamela Leece, Mina Tadrous, Ashley Smoke, Charlotte Munro, Tara Gomes

**Affiliations:** 1 Li Ka Shing Knowledge Institute, St. Michael’s Hospital, Toronto, Ontario, Canada; 2 Leslie Dan Faculty of Pharmacy, University of Toronto, Toronto, Ontario, Canada; 3 ICES, Toronto, Ontario, Canada; 4 Department of Family and Community Medicine, University of Toronto, Toronto, Ontario, Canada; 5 Campbell Family Mental Health Research Institute, Centre for Addiction and Mental Health, Toronto, Ontario, Canada; 6 Division of Addiction Medicine, Women’s College Hospital, Toronto, Ontario, Canada; 7 Centre for Addiction and Mental Health, Toronto, Ontario, Canada; 8 Department of Psychiatry, University of Toronto, Toronto, Ontario, Canada; 9 Public Health Ontario, Toronto, Ontario, Canada; 10 Dalla Lana School of Public Health, University of Toronto, Toronto, Ontario, Canada; 11 Women’s College Research Institute, Toronto, Ontario, Canada; 12 Ontario Drug Policy Research Network Lived Experience Advisory Group, Toronto, Ontario, Canada; 13 MAP Centre for Urban Health Solutions, St. Michael’s Hospital, Toronto, Ontario, Canada; 14 Institute of Health Policy, Management and Evaluation, University of Toronto, Toronto, Ontario, Canada; UCL: University College London, UNITED KINGDOM OF GREAT BRITAIN AND NORTHERN IRELAND

## Abstract

Early in the SARS-Cov-2 pandemic, modified clinical guidance recommended the provision of take-home methadone doses for those previously ineligible to facilitate social distancing. Following this change, studies reported improved treatment retention among patients granted expanded access to take-home doses. However, most patients resumed daily dispensed methadone within six months. Factors associated with the return to daily dispensed methadone remain unknown. Therefore, we conducted a population-based cohort study to identify patient and prescriber-related characteristics associated with return to daily dispensed methadone. Our study included all residents of Ontario, Canada who received daily dispensed methadone on March 21, 2020, and were then provided at least one take-home dose between March 22, 2020, and April 21, 2020. Follow-up time was divided into 14-day discrete time intervals. The primary outcome was return to daily dispensed methadone, defined as the first interval where a take-home dose was not dispensed. A multilevel discrete time survival model with a complementary log-log link function and random intercepts across prescribers to account for patient clustering by prescriber was used to approximate cause-specific hazard ratios. Within 26 weeks, 1,675 (58.5%) individuals were reverted to daily dispensed methadone. Person-level variables significantly associated with our primary outcome included occurrence of an emergency department visit during or before the interval of interest (HR = 1.27; 95% CI = 1.05, 1.55) and missed methadone dose(s) in the interval prior (HR = 1.59; 95% CI = 1.44, 1.76). Lastly, patients prescribed methadone by a high-volume (top 20^th^ percentile) opioid agonist treatment prescriber had an increased hazard of return to daily dispensed methadone compared to those prescribed methadone by a low-volume (50^th^ percentile) prescriber (HR = 1.44; 95% CI = 1.14, 1.83). While patient characteristics that may indicate clinical instability, such as recent history of missed methadone dose(s) were associated with return to daily dispensed methadone, prescriber OAT client volume was also be associated this outcome.

## Introduction

The North American opioid crisis is an ongoing public health concern that has claimed over 500,000 lives since 2016. Treatment with opioid agonist therapy (OAT; e.g., methadone, buprenorphine/naloxone) is recommended first line for people with opioid use disorder (OUD) and associated with a significantly reduced risk of mortality and morbidity among individuals who are retained on treatment, compared to those who discontinue or were not provided treatment [1–4]. Retention on OAT remains low due to several factors, including inadequate dosing [5,6], prolonged requirements for daily observed dosing [7], challenges attending daily pharmacy visits (i.e., work obligations, travel distance, stigma) [8–10], difficulties with transitions across care settings, increasing opioid tolerance associated with fentanyl use, and poor patient-provider relationships [11,12]. The high prevalence of OAT discontinuation is concerning due to the increased risk of opioid-related harm following disruptions in care [13,14].

The declaration of emergency for SARS-CoV-2 in March 2020 catalyzed rapid changes in methadone prescribing guidance across Canada and the United States (US). Although methadone recipients have historically required a minimum of 60–90 days in treatment with consistently negative urine toxicology tests before meeting eligibility for take-home doses, updated guidance recommended increased access to take-home doses to support physical distancing and reduce the risk of exposure to SARS-CoV-2 while maintaining continuity of OAT [15,16]. For example, federal authority in the US gave states permission to request up to 28 days of take-home medication for stable patients and up to 14 days for less stable patients. In Ontario, new guidance recommended provision of extended take-home doses based on the clinicians’ assessment of stability.

Following the release of pandemic specific OAT guidance in Ontario, the proportion of individuals receiving daily dispensed methadone declined from approximately one-third to one-quarter during the first month SARS-CoV-2 period [17]. However, the trend largely reverted to pre-pandemic levels by the November 2020 [17], despite evidence for no increased risk of opioid toxicity [18–20] and improved patient reported quality of life (e.g., increased time for employment/recreational activities, improved patient-provider relationship) among those granted access to take-home doses during the SARS-CoV-2 pandemic [21–23]. Therefore, we aimed to determine person and prescriber-related characteristics associated with return to daily dispensed methadone among individuals newly provided take-home doses during the first month of the SARS-CoV-2 pandemic.

## Materials and methods

### Ethics statement

ICES is designated a prescribed entity under Ontario’s Personal Health Information Privacy Act (PHIPA) and the Coroners Act. Section 45 of PHIPA authorizes ICES to collect personal health information without consent for the purpose of analyzing, compiling, or producing statistical information related to the management, evaluation, monitoring, resource allocation, or planning of all or part of the health system. Data used in this study is authorized under section 45 of Ontario’s Personal Health Information Protection Act and does not require review by a Research Ethics Board.

### Study design and setting

We conducted a population-based cohort study of Ontario residents who received daily dispensed methadone on March 21, 2020, and were subsequently dispensed at least one take-home methadone dose between March 22, 2020, and April 21, 2020. This study was pre-registered (doi: osf.io/7u2zr) and adheres to STROBE reporting guidelines [24].

### Data sources

We used data housed at ICES an independent, non-profit research institute whose legal status under Ontario’s health information privacy law allows it to collect and analyze health care and demographic data, without consent, for health system evaluation and improvement. The primary data source used was the Narcotics Monitoring System (NMS) to obtain information on all controlled substances (e.g., methadone) dispensed from community pharmacies regardless of payer. All datasets were linked using unique encoded identifiers and analyzed at ICES. See S1 Table and S1 Text for details on databases and data linkage methods, respectively.

### Cohort

We leveraged a pre-defined cohort of 2,865 individuals who were receiving daily dispensed methadone and were subsequently dispensed at least one take-home dose between March 22, 2020 to April 21, 2020 (i.e., month following release of pandemic specific take-home dose guidance) [18]. In summary, cohort creation consisted of two parts. First, we identified all individuals receiving daily dispensed methadone between our accrual window of February 22, 2020, to March 21, 2020. We included individuals treated with daily dispensed methadone (i.e., days’ supply equal to one) on March 21, 2020 (the day before release of the new guidance), who received methadone on at least 14 of the 28 days prior to March 21, 2020, and had a maximum day’s supply of 1 for all methadone doses dispensed in the week prior to March 22, 2020. Next our cohort was restricted to those provided at least one take-home methadone dose during our exposure window of March 22, 2020, to April 2020, with date the first take-home dose was dispensed defined as the index date. Lastly, we excluded those with missing patient identifiers, non-Ontario residents (i.e., without a valid Ontario healthcare number), those with missing age or sex, individuals younger than 18 or older than 105, those with a COVID-19 diagnosis prior to index, and those with no physician claim related to OAT or addiction medicine during our exposure window. Further details regarding cohort creation are published elsewhere [18]. For the purposes of our study, we accessed this data between June 1, 2024, to December 31, 2024.

### Outcomes

The primary outcome was return to daily dispensed methadone. Due to our use of pharmacy claims data, we were unable to determine the exact date the prescriber made the clinical decision to return their patient to daily dispensed methadone. Therefore, we divided person-time from index date to end of follow-up (i.e., observation of the primary outcome or censoring criteria) into a sequence of 14-day mutually exclusive discrete time intervals for each individual included in our cohort. In a given week, an individual may be provided both observed daily dispensed (i.e., administration of dose observed by a healthcare professional) methadone and take-home doses (e.g., provision of 2 take home doses and one observed dose on Friday to cover the patient over the weekend). Therefore, we defined return to daily dispensed methadone as the first 14-day interval where only observed doses (i.e., dispense records with a days’ supply equal to 1) were dispensed to ensure return to daily dispensed methadone. Censoring criteria included methadone discontinuation (defined as a treatment gap exceeding 14 days [13,14,18] with the last observed dispense date plus days’ supply defined as the discontinuation date), death, first dispense for a buprenorphine-containing product, hospital admission (all-cause), and end of follow-up (i.e., 182 days post index or 13^th^ interval). Because follow-up time was divided into 14-day intervals, the interval that included a censoring event was defined as censored. For example, if an individual met our definition for the outcome and a censoring criterion in the same interval, they were considered censored, as a complete interval observation was required to define return to daily dispensed methadone.

### Covariates

#### Patient-related variables.

We included demographic and clinical patient characteristics selected based on past literature [12,25] and clinical judgement. Demographic characteristics defined on index date included age, sex, neighbourhood income quintile and region of residence. We determined history of at least one emergency department (ED) or hospital visit for mental health or substance use disorders within the past two years (anxiety, deliberate self-harm, mood disorder, substance use disorder, trauma/stressor related or other), opioid related toxicity (past year), stimulant-related harmful use/dependence (past two years), alcohol use disorder (past three years) and invasive infection (past three years). Validated case definitions were used to define past diagnoses of human immunodeficiency virus (HIV), diabetes or chronic obstructive pulmonary disease at the index date. Health system utilization within the year prior to index was determined based on number of outpatient physician visits (related to opioid use disorder [OUD] and those not related to OUD), ED visits, and hospital visits. Medication use variables measured on index included use of buprenorphine/naloxone (past two years), milligram (mg) amount of methadone dispensed and presence of an active benzodiazepine prescription. Lastly, we identified records for any emergency department visit within the interval of interest or prior and occurrence of missed methadone dose(s) in the interval prior for each observed interval over our study period. See S2 Table for covariate definitions.

#### Prescriber variables.

We determined physician characteristics for the individual who prescribed the last take-home methadone dose dispensed for each interval of observation. For intervals where no take-home doses were dispensed, we ascertained characteristics for the physician who prescribed the last observed methadone record dispensed in the interval of interest. Physician characteristics included: specialty (family practice, emergency medicine, other), sex, years in clinical practice since medical school graduation and OAT client volume in the years prior to index (minimal [lowest 50^th^ percentile], moderate [51^st^ to 80^th^ percentile] and high [top 20^th^ percentile]).

### Statistical analysis

We used descriptive statistics to summarize person and prescriber variables at index date, reported overall and stratified by their initial prescriber’s (i.e., prescriber who provided take-home dose(s) observed at index) OAT client volume (i.e., minimal, moderate and high) in 2019 (i.e., year prior to index). We calculated p-values using student’s t-test (means) or sign test (medians) to detect significant differences across strata. A multilevel discrete time hazard model with a complementary log-log link function and random intercepts across prescribers was used to estimate cause-specific hazards ratios (HR) and 95% confidence intervals, while accounting for patient clustering among prescribers [26,27]. We considered methadone discontinuation as a competing risk, as those who discontinued methadone were no longer eligible to return to daily dispensed methadone. Therefore, we determined cause-specific hazard ratios, which represent the probability of failure due to the outcome of interest at a moment in time, given that no failure of any kind has occurred thus far [28].

We used a non-parsimonious model-building approach, where all covariates included in our model were selected a priori through consultation with the study team. Model diagnostics included tests for collinearity (using variance inflation factor) and the proportional hazard assumption using time interactions. For variables that significantly interacted with time, a time interaction was added to the model. No collinearity was detected between any variables. We used a type I error rate of 0.05 to determine statistical significance. All analyses were conducted using SAS version 9.4 (SAS Institute, Cary, North Caroline, USA).

### Involvement of people with lived experience

The Ontario Drug Policy Research Network’s opioid-related research is informed by a Lived Experience Advisory Group, comprised of individuals with lived and living experience using opioids who were consulted before, during, and after the study design process.

## Results

We identified 2,863 individuals who were newly provided take-home methadone dose(s) during the first month of the SARS-CoV-2 pandemic. Among our study cohort, the median age was 38 years (interquartile range, 32–47) and 63.8% were male (Table 1). Nearly one-half (48.0%) were prescribed their initial take-home dose by a physician with a high OAT client volume, and 84.3% were seen by a physician with family medicine training. In general, measures of comorbidity were similar when stratified by physician OAT client volume; however, use of outpatient healthcare services in the year prior to index differed across strata. Specifically, individuals provided their first take-home dose by a physician with a high OAT client volume had a higher number of OUD-related outpatient physician visits compared to those with a low OAT client volume prescriber (median OUD related visits: 25 vs. 22; p < 0.01). In contrast, those prescribed their first take-home dose by a physician with a low OAT client volume had a higher number of non-OUD related outpatient visits compared to those with a high-volume OAT prescriber (median non-OUD related visits: 7 vs. 4; p < 0.01).

**Table 1:** Baseline characteristics, reported overall and stratified by physician patient volume prescribed opioid agonist treatment.

	Overall (N = 2,863)	Physician Patient Volume^a^			p-value
		Low^b^ (N = 543, 19.0%)	Moderate^c^ (N = 946, 33.0%)	High^d^ (N = 1,374, 48.0%)	
**Patient-level Variables**					
**Age**					
Median (IQR)	38 (32-47)	40 (33-50)	38 (32-48)	37 (31-46)	<0.01
18-24	102 (3.6%)	20 (3.7%)	30 (3.2%)	52 (3.8%)	<0.01
25-44	1,853 (64.7%)	304 (56.0%)	615 (65.0%)	934 (68.0%)	
45-64	875 (30.6%)	210 (38.7%)	293 (31.0%)	372 (27.1%)	
65+	33 (1.2%)	9 (1.7%)	8 (0.8%)	16 (1.2%)	
**Sex**					
Female	1,036 (36.2%)	218 (40.1%)	323 (34.1%)	495 (36.0%)	0.07
Male	1,827 (63.8%)	325 (59.9%)	623 (65.9%)	879 (64.0%)	
**Rurality of Residence**					
Urban	2,558 (89.3%)	494 (91.0%)	831 (87.8%)	1,233 (89.7%)	0.34
Rural	288 (10.1%)	46 (8.5%)	110 (11.6%)	132 (9.6%)	
Missing	17 (0.6%)	<=5	<=5	9 (0.7%)	
**Income Quintile**					
1 (lowest)	1,119 (39.1%)	210 (38.7%)	348 (36.8%)	561 (40.8%)	0.14
2	676 (23.6%)	110 (20.3%)	243 (25.7%)	323 (23.5%)	
3	456 (15.9%)	91 (16.8%)	154 (16.3%)	211 (15.4%)	
4	370 (12.9%)	75 (13.8%)	119 (12.6%)	176 (12.8%)	
5 (highest)	222 (7.8%)	54 (9.9%)	77 (8.1%)	91 (6.6%)	
Missing	20 (0.7%)	<=5	<=5	12 (0.9%)	
**Emergency Department or Hospital Visit for Mental Health or substance use disorder (past 2 years)**					
Anxiety	105 (3.7%)	26 (4.8%)	34 (3.6%)	45 (3.3%)	0.28
Deliberate self-harm	124 (4.3%)	32 (5.9%)	44 (4.7%)	48 (3.5%)	0.06
Mood disorder	76 (2.7%)	13 (2.4%)	28 (3.0%)	35 (2.5%)	0.76
Substance use	480 (16.8%)	97 (17.9%)	176 (18.6%)	207 (15.1%)	0.06
Trauma/stressor-related disorder	103 (3.6%)	20 (3.7%)	40 (4.2%)	43 (3.1%)	0.37
Other	98 (3.4%)	21 (3.9%)	33 (3.5%)	44 (3.2%)	0.76
**Emergency Department or Hospital visit**					
For opioid-related toxicity (past year)	135 (4.7%)	29 (5.3%)	45 (4.8%)	61 (4.4%)	0.70
For stimulant-related harmful use/dependence (past 2 years)	238 (8.3%)	47 (8.7%)	83 (8.8%)	108 (7.9%)	0.70
Alcohol Use Disorder (past 3 years)	226 (7.9%)	52 (9.6%)	76 (8.0%)	98 (7.1%)	0.20
Invasive Infection (past 3 years)	252 (8.8%)	46 (8.5%)	94 (9.9%)	112 (8.2%)	0.31
**Diagnosis**					
Human Immunodeficiency Virus (HIV)	28 (1.0%)	6 (1.1%)	6 (0.6%)	16 (1.2%)	0.42
Diabetes	169 (5.9%)	41 (7.6%)	56 (5.9%)	72 (5.2%)	0.15
Chronic Obstructive Pulmonary Disease	306 (10.7%)	71 (13.1%)	103 (10.9%)	132 (9.6%)	0.08
**Health system utilization (past year)**					
Outpatient Physician Visits, median (IQR)					
Related to Opioid Use Disorder	24 (12-38)	22 (10-36)	23 (12-38)	25 (13-39)	<0.01
Non-Opioid Use Disorder Related	5 (1-15)	7 (2-16)	5 (1-16)	4 (1-13)	<0.01
≥ 1 Emergency Department Visit(s)	1,392 (48.6%)	249 (45.9%)	480 (50.7%)	663 (48.3%)	0.18
≥ 1 Hospital Visit(s)	299 (10.4%)	59 (10.9%)	102 (10.8%)	138 (10.0%)	0.80
**Buprenorphine/Naloxone Use (past 2 years)**	489 (17.1%)	93 (17.1%)	166 (17.5%)	230 (16.7%)	0.88
**Methadone Dose at Index**					
Median (IQR)	75 (50-100)	78 (50-100)	79 (50-105)	75 (49-100)	<0.01
0–60 mg	1,073 (37.5%)	197 (36.3%)	336 (35.5%)	540 (39.3%)	0.27
60> to 90 mg	822 (28.7%)	171 (31.5%)	268 (28.3%)	383 (27.9%)	
90> to 120 mg	683 (23.9%)	129 (23.8%)	239 (25.3%)	315 (22.9%)	
> 120 mg	285 (10.0%)	46 (8.5%)	103 (10.9%)	136 (9.9%)	
**Active Benzodiazepine Prescription at Index**	333 (11.6%)	79 (14.5%)	120 (12.7%)	134 (9.8%)	0.01
**Index Period Values for Time-Varying Patient-Level Variables**					
Emergency Department Visit (interval of interest or prior)	173 (6.0%)	30 (5.5%)	59 (6.2%)	84 (6.1%)	0.85
Missed Methadone Dose in Interval Prior	1,221 (42.6%)	218 (40.1%)	391 (41.3%)	612 (44.5%)	0.13
**Index Values for Time-Varying Prescriber-Level Variables**					
**Physician Specialty**					
Family Medicine	2,414 (84.3%)	446 (82.1%)	799 (84.5%)	1,169 (85.1%)	0.28
Other	449 (15.7%)	97 (17.9%)	147 (15.5%)	205 (14.9%)	
**Sex**					
Female	610 (21.3%)	143 (26.3%)	240 (25.4%)	227 (16.5%)	<0.01
Male	2,253 (78.7%)	400 (73.7%)	706 (74.6%)	1,147 (83.5%)	
**Years in Clinical Practice**					
0–5	101 (3.5%)	7 (1.3%)	54 (5.7%)	40 (2.9%)	<0.01
6–10	141 (4.9%)	38 (7.0%)	21 (2.2%)	82 (6.0%)	
11 or more	2,621 (91.5%)	498 (91.7%)	871 (92.1%)	1,252 (91.1%)	

^a^Prescriber identified at index date.

^b^Opioid agonist treatment client volume within the lowest 50th percentile.

^c^Opioid agonist treatment client volume within the 51st to 80th percentile.

^d^Opioid agonist treatment client volume within the top 20th percentile.

Overall, 1,675 (58.5%) individuals returned to daily dispensed methadone, while 318 (11.1%) discontinued methadone within 26 weeks of the index date (see S3 Table for censoring criteria frequencies). Conditional on random effects, and controlling for all covariates, patient-related variables significantly associated with an increased hazard of return to daily dispensed methadone included occurrence of an emergency department visit during the interval of interest or in the interval immediately before (HR = 1.27; 95% CI = 1.05, 1.55) and missed methadone dose(s) in the interval immediately prior (HR = 1.59; 95% CI = 1.44, 1.76; Table 2). For every 10 visits to an outpatient physician for an OUD-related reason in the year prior to index, the hazard of return to daily dispensed methadone increased by 5% (95% CI = 1.01, 1.10). Patient-related variables significantly associated with a lower hazard of return to daily dispensed methadone included a prior diagnosis of HIV (HR = 0.40; 95% = 0.21, 0.78) or diabetes (HR = 0.75; 95% CI = 0.59, 0.95). Lastly, patients prescribed methadone by a physician with 6–10 years of clinical experience (HR = 1.91; 95% CI = 1.07, 3.44) and those treated by a physician with a high-volume of OAT clients (HR = 1.44; 1.14, 1.83) had an increased hazard of returning to daily dispensed methadone compared to those with zero to five and a low OAT client volume, respectively.

**Table 2:** Result from discrete time mixed effects model: association between patient and prescriber-related variables and return to daily dispense methadone.

	Approximate Adjusted Hazard Ratio (95% CI)
**Person-level Variables**	
**Age (years)**	
15–24 (ref)	1.0
25-44	1.17 (0.88, 1.56)
45-64	1.15 (0.85, 1.55)
65+	0.82 (0.44, 1.51)
**Sex**	
Female (ref)	1.0
Male	1.01 (0.91, 1.12)
**Region of residence**	
Urban (ref)	1.0
Rural	0.94 (0.78, 1.12)
Missing	1.11 (0.29, 4.26)
**Income quintile**	
1	1.20 (0.97, 1.47)
2	1.05 (0.85, 1.30)
3	1.17 (0.93, 1.47)
4	1.09 (0.86, 1.37)
Missing	0.99 (0.30, 3.25)
5 (ref)	1.0
**Mental Health Related Emergency Department/Hospital Visit (past 2 years)**	
Anxiety	
No (ref)	1.0
Yes	1.19 (0.89, 1.57)
Deliberate self-harm	
No (ref)	1.0
Yes	0.95 (0.72, 1.26)
Mood disorder	
No (ref)	1.0
Yes	1.10 (0.80, 1.51)
Substance abuse	
No (ref)	1.0
Yes	1.08 (0.91, 1.28)
Trauma/stressor-related disorder	
No (ref)	1.0
Yes	1.09 (0.79, 1.49)
Other	
No (ref)	1.0
Yes	0.89 (0.65, 1.22)
**Emergency Department or Hospital Visit**	
For opioid-related toxicity (past year)	
No (ref)	1.0
Yes	1.14 (0.88, 1.47)
For stimulant-related harmful use/dependence (past 2 years)	
No (ref)	1.0
Yes	1.15 (0.93, 1.42)
Invasive Infection (past 3 years)	
No (ref)	1.0
Yes	1.73 (1.30, 2.31)
Invasive Infection in the past 3 year*interval	0.93 (0.88, 0.99)
**Diagnosis**	
Alcohol Use Disorder (past 3 years)	
No (ref)	1.0
Yes	0.98 (0.79, 1.21)
Human Immunodeficiency Virus	
No (ref)	1.0
Yes	0.40 (0.21, 0.78)
Diabetes	
No (ref)	1.0
Yes	0.75 (0.59, 0.95)
Chronic Obstructive Pulmonary Disease	
No (ref)	1.0
Yes	0.99 (0.82, 1.19)
**Health System Visits in the past year (per 10 visits)**	
Outpatient Physician Visit Related to Opioid Use Disorder	1.05 (1.01, 1.10)
Outpatient Physician Visit Not Related to Opioid Use Disorder	0.97 (0.93, 1.02)
Emergency Department Visits	0.89 (0.67, 1.18)
Hospital Visits	0.62 (0.19, 2.03)
**Buprenorphine/Naloxone Use (past 2 years)**	
No (ref)	1.0
Yes	0.95 (0.82, 1.09)
**Methadone Dose**	
At baseline	
0–60 mg (ref)	1.0
60> to 90 mg	0.98 (0.80, 1.20)
90> to 120 mg	1.13 (0.91, 1.40)
> 120 mg	0.71 (0.52, 0.97)
Methadone dose at baseline*interval	
0–60 mg (ref)	1.0
60> to 90 mg	1.03 (0.99, 1.06)
90> to 120 mg	1.00 (0.96, 1.04)
> 120 mg	1.07 (1.02, 1.13)
**Active Benzodiazepine Prescription at Index**	
No (ref)	1.0
Yes	0.99 (0.84, 1.17)
**Time-Varying Patient-Level Variables**	
Emergency Department Visit (interval of interest or prior)	
No (ref)	1.0
Yes	1.27 (1.05, 1.55)
Missed Methadone Dose (Interval Prior)	
No (ref)	1.0
Yes	1.59 (1.44, 1.76)
**Time Interval**	0.92 (0.90, 0.95)
**Prescriber-Level Variables**	
**Physician Specialty**	
Family Medicine (ref)	1.0
Emergency Medicine	1.53 (0.80, 2.94)
Other	1.28 (0.99, 1.65)
Missing	0.06 (0.00, 2151976848417.69)
**Physician Sex**	
Female	1.0
Male	0.94 (0.75, 1.16)
**Years in Clinical Practice**	
0–5 (ref)	1.0
6–10	1.91 (1.07, 3.44)
11 or more	1.59 (0.98, 2.58)
**Opioid Agonist Treatment Client Volume**	
Low (lowest 50th percentile)	1.0
Moderate (51st to 80th percentile)	1.24 (0.98, 1.57)
High (top 20th percentile)	1.44 (1.14, 1.83)

*Time interaction.

When testing the proportional hazards assumption, violations were observed among the following baseline person-related variables: history of invasive infection and methadone dose dispensed on index. After taking time since index date into consideration, the effect of prior history for invasive infection was no longer significantly associated with return to daily dispensed methadone (estimate at interval 1: HR = 1.62, 95% CI = 0.98 to 2.66; estimate at interval 4: HR = 1.30, 95% CI: 0.54 to 3.16). Although individuals dispensed methadone doses ≥ 120mg at index initially had a lower hazard of return to daily dispensed methadone compared to those dispensed doses ≤60mg (estimate at interval 1: HR = 0.76; 95% CI = 0.60, 0.97), this relationship dissipated over time (estimate at interval 4: HR = 0.94; 95% CI = 0.81, 1.09).

## Discussion

In the 26 weeks following the initial provision of take-home doses to individuals previously receiving daily dispensed methadone—a change in clinical practice influenced by enactment of SARS-CoV-2 related state of emergency and release of revised take-home OAT guidelines—we found that only 3 out of 10 people-maintained access to take-home dose(s). We identified a number of patient-related characteristics associated with this change that may indicate inconsistent engagement with treatment and clinical instability, including recent history of missed methadone dose(s) and emergency department visit(s) – factors that may warrant return to daily dispensed methadone. Individuals prescribed methadone by a high-volume OAT prescriber, who typically operate in large OAT clinics, were less likely to sustain take-home doses, independent of their clinical history. Given the positive impact of take-home doses on treatment retention and patient satisfaction [7,18], and the potential risk of treatment disruption and opioid-related harm when they are removed [7,29], equitable access to take-home doses must be prioritized across all methadone prescribers.

The revised pandemic guidance, which remained in effect for the duration of our study period, de-emphasized abstinence and contingency management as a criteria for provision for take-home doses [16]. Instead, decisions regarding provision of take-home doses were based on signs of clinical instability such as a recent overdose, unstable psychiatric comorbidity, or high-risk use of unregulated substances [16]. Similar guidance was also recommended across other jurisdictions, including the US, where the Substance Abuse and Mental Health Services Administration recommended up to 28 days of take-home doses among stable patients and up to 14 days among less stable patients [15]. This revised criteria for take-home doses aligns with our findings of a positive association between return to daily dispensed methadone and history of a recent ED visit or missed methadone doses, factors that may indicate clinical instability. Of note, signs of clinical instability (e.g., missed methadone doses) may also be influenced by systemic barriers to treatment engagement often experienced by patients with OUD, including limited access to methadone dispensing pharmacies, experiences of structural violence and unstable housing. Since the release of pandemic-specific guidance organizations across Canada and the US, revised guidance that promotes ongoing flexibility in the provision of take-home methadone doses has been released [25,30,31]. These updated guidelines remove rigid requirements for 60-days of daily dispensed methadone prior to provision of take-home doses and place a greater emphasis on the prescriber’s clinical judgment [25,30,31].

Despite continued efforts for expanded access to take-home doses, we found that - independent of clinical characteristics – provision of methadone by a high-volume (top 20^th^ percentile) OAT prescriber was associated with return to daily dispensed methadone relative to individuals with a low-volume OAT prescriber. Various factors may influence a physician’s willingness to prescribe take-home doses, including concerns of diversion, knowledge of clinical guidelines, and relationship with the patient [22,32]. In Ontario, high-volume OAT prescribers typically operate in stand-alone OAT clinics. While a majority of these prescribers are trained in family medicine, their practice is generally OAT-focused [33]. On average, high-volume methadone prescribers treat 97 OAT patients per day [33]. Due to time constraints, these visits may be brief in duration, which may make it difficult to establish a strong patient-prescriber relationship [34]. It is possible that decision-making surrounding sustained delivery of take-home doses among high-volume OAT prescribers reflects protocolized and risk-averse (e.g., requirements for negative urine drug screens) care models. Moreover, while making current guidance more open-ended may support patient-centered decision-making, a recent systematic review of Canadian OUD guidelines attributed inconsistencies and the vague definitions for “stability” on OAT as a driver for inequitable access to take-home doses [12]. Future research should focus on developing individualized risk assessment tools to help identify patients who can safely manage take-home doses and to promote equitable access to methadone prescribing across practice settings.

It is likely that people prescribed methadone by a low- to moderate-volume OAT prescriber visited a primary care or comprehensive addiction medicine clinic, where they receive care for OUD and other existing comorbidities. This aligns with our findings, where patients who received their first take-home dose from a low-volume OAT prescriber had a greater number of outpatient physician visits for non-OUD concerns in the year prior, compared with those treated by high-volume prescribers. Such integrated care has been associated with improved patient satisfaction and patient-prescriber relationship [35,36]. Alternatively, because high-volume prescribers have more experience managing patients with OUD, they may be more skilled in identifying high-risk patients who are not equipped to safely manage take-home doses, resulting in a greater number of patients returning to daily dispensed methadone. Future research should evaluate the differences in quality of care provided by OAT prescribers across different practice settings and further investigate patient outcomes for those who maintained access to take-home doses over a longer period of time.

This study has several limitations. Although we captured a number of clinical variables to determine patient stability, we were unable to capture all factors that may influence a clinician’s decision surrounding take-home doses, such as presence of ongoing drug use (e.g., exposure to multiple substance) or implicit bias towards certain sub-populations (e.g., racialized groups). Additionally, social determinants (e.g., job security, stable housing, travel requirements to a methadone dispensing pharmacy) of health that may influence a patient’s ability to sustain take-home doses within the framework of the current care model were not measured. While we attempted to measure individual income using the income quintile variable, this measure is based on neighborhood income derived from census data. Third, we were unable to determine the exact date the decision to revert the patient to daily dispensed methadone was made, and which specific physician made this decision, placing our study at risk of outcome and prescriber misclassification. However, by making the methodological decision to select either the prescriber who provided take-home doses or the last methadone prescriber within a given interval it is likely we selected the prescriber most relevant to the decision-making process surrounding the return to daily dispensed methadone in our models. Furthermore, 56% of people in our cohort saw one prescriber during follow-up and less than 15% saw four or more prescribers, meaning that this misclassification was likely minimal. Fourth, our study was limited to Ontario residents registered the provincial health plan, which may further limit the generalizability of our findings to jurisdictions across and outside of Canada due to differences in government-sponsored health insurance. Further, our findings may exclude new Ontario residents who have not yet registered for the provincial health plan, although this number is expected to be small. Finally, we used discrete 14-day intervals to more accurately capture the time point the decision to return to daily dispensed methadone was made versus discontinuation, however it is possible that a patient received take-home doses and was reverted to daily dispensed methadone within the same interval.

## Conclusion

Access to take-home doses is associated with improved treatment retention and patient’s quality of life due to increased autonomy of their daily schedule [7,18]. While patient characteristics that may indicate clinical instability, such as recent history of missed methadone dose(s) and ED visits were associated with - and may warrant - return to daily dispensed methadone, prescriber OAT client volume may also be associated this outcome. Currently, a majority of methadone is delivered through high-volume prescribers [33], who typically operate in large scale OAT clinics. Coordination and integration of care between OAT clinics and primary care must be prioritized to support equitable access to take-home methadone doses.

## Supporting information

S1 Table Description of all linked administrative databases used in the study. (DOCX)

S2 Table Definitions for each covariate included in statistical model. (DOCX)

S3 Table Censoring criteria for return to daily dispense outcome. (DOCX)

S1 Text Database linkage methods. (DOCX)
