# Supplementary material for: Patient and Prescriber characteristics associated with return to daily-dispense methadone: A multilevel cohort study
Source: PLOS Ment Health. 2025 Nov 7;2(11):e0000442. doi: 10.1371/journal.pmen.0000442 (PMC12798419; doi:10.1371/journal.pmen.0000442)
Supplement: S1 Table — (DOCX) [file pmen.0000442.s001.docx]

**S1 Table.** Description of All Linked Administrative Databases Used in the Study

| **Database** | **Description** |
| --- | --- |
| Narcotics Monitoring System (NMS) | Captures all prescriptions for controlled substances dispensed from community pharmacies in Ontario, regardless of insurance status. This database was used to identify opioid agonist therapy dispensations. |
| Registered Persons Database (RPDB) | Captures demographic and vital status characteristics for everyone eligible for the publicly-funded Ontario Health Insurance Plan (OHIP). |
| Canadian Institute for Health Information (CIHI) Discharge Abstract Database (DAD) | Captures details on diagnoses and procedures for all inpatient hospital stays in Ontario. |
| CIHI National Ambulatory Care Reporting System (NACRS) | Captures details on diagnoses and procedures for all emergency department visits in Ontario. |
| CIHI Ontario Mental Health Reporting System (OMHRS) | Captures details on diagnoses and procedures for all inpatient stays in mental health hospitals in Ontario. |
| Ontario Health Insurance Plan (OHIP) Database | Captures outpatient care using billing information for all services covered by OHIP in Ontario. |
| ICES Physician Database (IPDB) | Captures information on physcians eligible to receive payment from the OHIP, such as demographics, training, and practice location. |
| Ontario Diabetes Database | This database uses a validated definition to identify diagnosis dates fo all people with diabetes in Ontario (90.0% sensitivity, 97.7% specificity). |
| Chronic Obstructive Pulmonary Disease (COPD) Validated Database | This database uses a validated definition to identify diagnosis dates for all people with COPD in Ontario (85.0% sensitivity, 78.4% specificity). |
| HIV Validated Database | This database uses a validated definition to identify diagnosis dates for all people with HIV in Ontario (96.2% sensitivity, 99.6% specificity). |
| Drug-Alcohol Related Death Database | Contains details from investigations for all deaths occurring in Ontario where an investigating coroner has determined opioids to be direct contributors to death. |
