# Supplementary material for: Patient and Prescriber characteristics associated with return to daily-dispense methadone: A multilevel cohort study
Source: PLOS Ment Health. 2025 Nov 7;2(11):e0000442. doi: 10.1371/journal.pmen.0000442 (PMC12798419; doi:10.1371/journal.pmen.0000442)
Supplement: S2 Table — (DOCX) [file pmen.0000442.s002.docx]

**S2 Table.** Definitions for each covariate included in statistical model

| **Variable** | **Database** | **Definition** |
| --- | --- | --- |
| **Patient-related covariates** (measured at baseline) | | |
| Age | RPDB | Age at index using birth date in RPDB |
| Sex | RPDB | Sex as recorded in RPDB at index |
| Rurality of residence | RPDB | Rurality of residence using person’s postal code at index |
| Neighbourhood income quintile | RPDB | Neighbourhood income quintile using person’s postal code at index |
| Mental Health Related Emergency Department/Hospital Visit (past 2 years) | NACRS, DAD, OMHRS | Any mental health and addictions (based on main/most responsible diagnoses) with the following diagnosis codes in the 2 years prior to index date:  **Anxiety Disorder**  ICD-9 Codes: 293.84, 300, 300.0x, 300.2x, 309.21, 313.23. DSM Provisional=5  ICD-10 Codes: F06.4, F40, F41, F93.0, F93.1, F93.2, F94.0  **Deliberate self-harm**  ICD-10 Codes: X60-X84, Y10-Y19, Y28  **Mood disorder**  ICD-9 Codes: 293.83, 296.x (all 296 codes), 300.4x, 301.13, 311.x, 625.4. DSM Provisional =3, 4  ICD-10 Codes: F06.3, F30.x-F34.x, F38.x, F39.x, F53.0  **Substance abuse**  ICD-9 Codes: 291.x, 292.x, 303.x, 304.x, 305.x, 312.31, DSM Provisional=16  ICD-10 Codes: F10, F11, F12, F13, F14, F15, F16, F17, F18, F19, F55, F63.0  **Trama/stressor-related disorder**  ICD-9 Codes: 308.3x, 309, 309.0x, 309.24, 309.28, 309.3x, 309.4x, 309.81, 309.89, 309.9x, 313.89. DSM Provisional =7  ICD-10 Codes: F43.x, F94.1, F94.2  **OTHER:**  Schizophrenia Spectrum and Other Psychotic Disorders:  ICD-9 Codes: 293.81, 293.82, 295.x, 297.x, all 298.x, DSM Provisional=2  ICD-10 Codes: F06.0, F06.1, F06.2, F20, F22-F29, F53.1  OCD & related disorders:  ICD-9 Codes: 300.3x, 300.7x, 312.39, 698.4x. DSM Provisional =6  ICD-10 Codes: F42.x, F45.2, F63.3  Personality disorders:  ICD-9 Codes: 301, 301.0x, 301.2x, 301.4x, 301.5x, 301.6x, 301.7x, 301.81-3, 301.89, 301.9x 310.1. DSM Provisional =18  ICD-10 Codes: F07.0, F21, F60, F61, F62. F68, F69  Other:  ICD-10 Codes: Any other diagnosis code between F06 and F99 not included in the categories above, excluding dementia and delirium-related diagnoses. |
| Opioid-related toxicity | DAD, NACRS | ED visit or inpatient hospitalization with diagnosis of opioid poisoning using codes below in the 1 year prior to index date  ICD-10 Codes: T40.0, T40.1, T40.2, T40.3, T40.4, T40.6 |
| Stimulant-related harmful use/dependence | DAD, NACRS | ED visit or inpatient hospitalization related to stimulant use or dependence using the codes below in the 2 years prior to index date  ICD-10 Codes: F14, F15, T43.6, T40.5 |
| Invasive infection | DAD, NACRS | ED visit or inpatient hospitalization related to stimulant use or dependence using the codes below in the 3 years prior to index date  **Osteomyelitis**  ICD-10 Codes: M86*, M899  **Endocarditis**  ICD-10 Codes: B376, I33, I34*, I35, I36, I37, I38, I39  **Sepsis**  ICD-10 Codes: A40, A41, I269, I400, R572, R651, R659  **Skin and Soft Tissue Infection**  ICD-10 Codes: I80, L97, L988, M793, A480, G06, G09, K630, K650, K750, L02, L03, M5402, M726, N10, R02 |
| Alcohol Use Disorder | OHIP, DAD, NACRS | Physician or hospital related visit for alcohol use disorder using codes below in the three years prior to index date (any diagnosis type):  ICD-10 Codes: F10 Z50.2 Z71.4 Z86.40 Z72.1 K70 G31.2 G62.1 G72.1 I42.6 K29.2 K86.0 T51.0 E24.4 K85.2  OHIP Diagnosis Codes: 291 303 |
| Human Immunodeficiency Virus | HIV validated database | Diagnosis of HIV prior to index date using validated definition:  Citiation: Antoniou T, Zagorski B, Loutfy MR, Strike C, Glazier RH. Validation of case-finding algorithms derived from administrative data for identifying adults living with human immunodeficiency virus infection. PLoS One. 2011;6(6):e21748. |
| Diabetes | Diabetes validated database | Diagnosis of diabetes prior to index date using validated database.  Citation: Lipscombe LL, Hwee J, Webster L, Shah BR, Booth GL, Tu K. Identifying diabetes cases from administrative data: a population-based validation study. BMC Health Serv Res. 2018;18(1):316. |
| Chronic Obstructive Pulmonary Disease | COPD validated database | Diagnosis of COPD prior to index date using validated database.  Citation: Gershon AS, Wang C, Guan J, Vasilevska-Ristovska J, Cicutto L, To T. Identifying individuals with physician diagnosed COPD in health administrative databases. COPD. 2009;6(5):388-394. |
| Number of physician visits for opioid-use disorder | OHIP | Number of OHIP billings with the following feecodes in the year prior to index date:  OHIP Feecode: A957, K680, K682, K683 |
| Number of outpatient visits for non OUD-related reasons | OHIP | Number of OHIP billings overall, excluding billings related to OUD (as defined above) in the year prior to index date. |
| Number of ED visits | NACRS | Number of unique ED visits in the year prior to index date. All ED visits are considered, regardless of diagnoses or procedures identified. |
| Number of inpatient hospitalizations | DAD | Number of unique inpatient hospital stays in the year prior to index date. All hospitalizations are considered, regardless of diagnoses or procedures identified. |
| Past buprenorphine/ naloxone use | NMS | Defined as any buprenorphine/naloxone dispense in the 2 years prior to index date. |
| Daily methadone dose | NMS | measured as total quantity * strength /days supply and reported in milligrams, based on methadone dispense record identified on index |
| Active benzodiazepine prescription at index | NMS | A prescription for a benzodiazepine with duration overlapping index date |
| **Patient-related covariates** (Time-varying) | | |
| Emergency department visit in the interval of interest or prior | NACRS | Any ED visit in the interval of interest or prior throughout follow-up. |
| Missed methadone dose in the interval prior | NMS | Presence of a missed dose in the interval prior throughout follow-up, defined as observation of <14 methadone dispenses. |
| **Prescriber-related covariates** | | |
| Prescriber type | NMS, IPDB | Prescriber identified on the methadone dispense record of interest throughout follow-up. |
| OAT prescriber volume | NMS, IPDB | Defined based on the distribution of unique OAT clients in 2019 per prescriber identified in our study cohort. Time-updated for each prescriber of interest throughout follow-up |
| Physician Sex | NMS, IPDB | Sex as recorded in IPDB for physician of interest. |
| Years in clinical practice | NMS, IPDB | Defined as number of years between year of index-date minus date of medical school graduation for each physician of interest. |
