# Supplementary material for: Patient and Prescriber characteristics associated with return to daily-dispense methadone: A multilevel cohort study
Source: PLOS Ment Health. 2025 Nov 7;2(11):e0000442. doi: 10.1371/journal.pmen.0000442 (PMC12798419; doi:10.1371/journal.pmen.0000442)
Supplement: S3 Table — (DOCX) [file pmen.0000442.s003.docx]

**S3 Table.** Censoring Criteria for Return to Daily Dispense Outcome

| **Censoring Criteria** | **Number of individuals censored** |
| --- | --- |
| Discontinuation | 318 (11.1%) |
| Hospitalization | 81 (2.8%) |
| Switch to buprenorphine | 33 (1.2%) |
| Death | <6 |
| End of follow-up | 754 (26.3%) |
