# Supplementary material for: Patient and Prescriber characteristics associated with return to daily-dispense methadone: A multilevel cohort study
Source: PLOS Ment Health. 2025 Nov 7;2(11):e0000442. doi: 10.1371/journal.pmen.0000442 (PMC12798419; doi:10.1371/journal.pmen.0000442)
Supplement: S1 Text — (DOCX) [file pmen.0000442.s004.docx]

**S1 Text.** Database linkage methods.

ICES links all databases using unique encoded identifiers derived through deterministic linkage of an individual’s publicly funded Ontario Health Insurance Plan (OHIP) card number fuzzy matching algorithms using other patient identifiers when OHIP card numbers are not available. By using combination of deterministic linkage and fuzzy match algorithms, the multi-pass linkage strategy is maintained by passing unlinked records in subsequent linkage comparison cycles. This method uses personal identifiers of given names, surnames, date of birth, death date (if applicable) and sex. In each pass, the pool of possible matched pairs is created by merging on low sensitive match codes with different conditions. Then, using a rule-based approach, possible pairs are examined, flags are assigned, and survival rules are applied to select the best matched records. In 2021 (the year our study was conducted), 99.58%, 97.79%, 97.43% and 100% of records in the Narcotics Monitoring System, Discharge Abstract Database, National Ambulatory Care Reporting System, and OHIP database, respectively had a valid unique code identifier, highlighting the strength and validity of data linkage across healthcare databases housed at ICES.
